# Comparison of discrimination and calibration performance of ECG-based machine learning models for prediction of new-onset atrial fibrillation

**DOI:** 10.1186/s12874-023-01989-3

**Published:** 2023-07-22

**Authors:** Giovanni Baj, Ilaria Gandin, Arjuna Scagnetto, Luca Bortolussi, Chiara Cappelletto, Andrea Di Lenarda, Giulia Barbati

**Affiliations:** 1 Department of Mathematics and Geosciences, University of Trieste, Trieste, Italy; 2 Department of Medical Sciences, Biostatistics Unit, University of Trieste, Trieste, Italy; 3 Cardiovascular Center, Territorial Specialistic Department, University Hospital and Health Services of Trieste, Trieste, Italy

**Keywords:** Atrial fibrillation, Prediction, Calibration, Machine learning, Deep learning

## Abstract

**Background:**

Machine learning (ML) methods to build prediction models starting from electrocardiogram (ECG) signals are an emerging research field. The aim of the present study is to investigate the performances of two ML approaches based on ECGs for the prediction of new-onset atrial fibrillation (AF), in terms of discrimination, calibration and sample size dependence.

**Methods:**

We trained two models to predict new-onset AF: a convolutional neural network (CNN), that takes as input the raw ECG signals, and an eXtreme Gradient Boosting model (XGB), that uses the signal’s extracted features. A penalized logistic regression model (LR) was used as a benchmark. Discrimination was evaluated with the area under the ROC curve, while calibration with the integrated calibration index. We investigated the dependence of models’ performances on the sample size and on class imbalance corrections introduced with random under-sampling.

**Results:**

CNN's discrimination was the most affected by the sample size, outperforming XGB and LR only around *n* = 10.000 observations. Calibration showed only a small dependence on the sample size for all the models considered.

Balancing the training set with random undersampling did not improve discrimination in any of the models. Instead, the main effect of imbalance corrections was to worsen the models’ calibration (for CNN, integrated calibration index from 0.014 [0.01, 0.018] to 0.17 [0.16, 0.19]).

The sample size emerged as a fundamental point for developing the CNN model, especially in terms of discrimination (AUC = 0.75 [0.73, 0.77] when *n* = 10.000, AUC = 0.80 [0.79, 0.81] when *n* = 150.000). The effect of the sample size on the other two models was weaker. Imbalance corrections led to poorly calibrated models, for all the approaches considered, reducing the clinical utility of the models.

**Conclusions:**

Our results suggest that the choice of approach in the analysis of ECG should be based on the amount of data available, preferring more standard models for small datasets. Moreover, imbalance correction methods should be avoided when developing clinical prediction models, where calibration is crucial.

**Supplementary Information:**

The online version contains supplementary material available at 10.1186/s12874-023-01989-3.

## Background

In the last few years, there has been a growing interest in the potential diagnostic value provided by electrocardiogram (ECG) signals. ECG waveform is one of the most extensively studied physiological signals to evaluate the condition of the heart, in which several waves as P, R, and T, are key to determining the type of rhythm. The interpretation of ECGs is complex and requires inspection by highly trained clinicians. However, numerous studies have shown that computer-aided methods based on ECG data represent a promising tool for the analysis and identification of cardiovascular diseases [1].

One example is the prediction of atrial fibrillation (AF), the most common supraventricular arrhythmia in the general population. AF is a relevant risk factor for stroke, however, it is often asymptomatic and not recognized. Thus, the identification of patients at high risk of future development of AF represents a major challenge. The way AF detection and prediction are evolving with the availability of new predictive tools is well described in a review carried out by Siontis et al. [2]. The development of tools to predict AF from routine and low-cost exams such as ECG would be an important step toward the active targeting of patients at risk, a task for which clinical risk scores and electronic health record-based tools have shown limited power [3].

The 12-lead ECG is a rapid, cost-effective cardiological exam that is routinely performed at different levels of point-of-care, from hospitals to clinics and ambulatory centres, generating a massive number of digital traces. As for other types of Big Data in the healthcare context, a major role in their analysis may be played by Artificial Intelligence (AI) systems, which can be easily fed with hundreds of thousands of observations. Two main approaches can be distinguished for the development of diagnostic models based on ECG. One approach involves the analysis of ECG features. Automated ECG interpretation is not a new concept, and algorithms that provide ECG interpretations have been around for a long time (in many cases code is proprietary and not disclosed). Such computer programs usually work in separate stages, including signal pre-processing, beat identification, correction, computation of average beats, and identification of fiducial points from which ECG measurements are extracted. Such measurements rely on knowledge-driven markers (like QRS, ST-segment elevation, T-wave changes) reflecting the clinical knowledge of heart activity, and can be then used to define criteria and rules for a diagnostic evaluation by physicians. In addition to human evaluation, in the last years ECG features, which can vary in number and type depending on the program employed, have been used to feed ML methods for tabular data to derive a diagnostic model [4–7].

The second approach consists in developing end-to-end prediction models that do not require feature extraction. This strategy involves the direct analysis of the digital ECG waveform to obtain the probability of a specific class in the classification of interest and deep learning (DL) based neural networks have demonstrated to be able to achieve good results. Despite DL models being black boxes and requiring the application of explainability techniques to investigate their prediction mechanism, this AI method for ECG analysis is being increasingly explored for its ability to detect subtle and non-linear interrelated variations along the signal [8]. The most common DL architectures used for analyzing ECGs are convolutional neural networks (CNN), a specialized kind of neural network for pattern recognition in time series and image data [9]. These networks can be thought of as having two sequential components: in the first layers, a set of convolutional filters allows us to extract patterns and key features from the signal, while in the second part, these extracted features are combined and used to make a prediction. Notice that the specific weights of the filters to be applied and the relative features extracted are automatically learnt by the network in the training process. It has been recently shown that the performance of a CNN in classifying arrhythmia from ECG can exceed that of cardiologists with average experience [10, 11]. Besides this classification task, CNNs have already shown good performances in predicting the new onset of AF (see Raghunath et al. [12] for AF prediction within 1year, and Attia e al. [13] for the identification of electrocardiographic signature of AF immediately prior to diagnosis). All these works reported quite good values of discrimination accuracy, but no information was available about the calibration of the estimated probabilities.

We stress that the diagnostic/classification task is quite different from the prediction task in epidemiological studies: classification is best used to identify the presence of an outcome/condition in the context of case–control studies. On the contrary, in the context of cohort studies when subjects are selected as initially free from the outcome and are then followed in time until they will (or will not) develop the outcome, usually observed in a minority of subjects, modelling tendencies (i.e., probabilities) is key [14]. The common approach of balancing events/non-events cases before applying ML/DL algorithms, based on the perception that this procedure can improve performance, seems not advisable in the prediction context [15]. The consequence of balancing could be that the algorithm trained to “predict” a 1/2 incidence of events will not be applicable to a population with a 1/1000 incidence. Subsequent calibration procedures are then needed in order to correct this issue [16]. Since the low incidence of new-onset AF in our population, the possible impact of balancing was an issue that we wanted to explore in the context of AF prediction.

The main goal of the present research was the development of a predictive model for a binary outcome based only on ECG information by comparing different methods: an ML algorithm on signal features and a DL approach on raw signals. Penalized logistic regression was used as a benchmark method. In this framework, AF represents a case study and this research does not claim to propose a prediction tool suitable for the clinical practice. Instead, our effort is aimed to extensively analyze the performance of the two approaches in terms of discrimination and calibration taking into account varying sample sizes and degrees of balance between the events and the censored cases. In particular, our research was based on different hypotheses: a) DL models based on the raw ECG signals could potentially outperform algorithms working on ECG features when the training set is large; b) the use of under-sampling to handle class imbalance does not improve discriminative performance and could instead produce miscalibrated predictions.

## Methods

### Data

We included all subjects aged > 30 years with at least one standard 10-s, 12-lead ECG acquired at the Cardiovascular Department of Azienda Sanitaria Universitaria Giuliano Isontina (ASUGI) in Trieste, between February 2, 2007, and December 31, 2020. ECGs were recorded at a frequency of 1 kHz using the Mortara™ devices ELI230 and ELI250, and then resampled at 500 Hz for computational reasons. By linking the ECG exams with the Electronic Health Records of the Regional Epidemiological Repository of Friuli Venezia Giulia region (Italy), we could integrate them with all the cardiological clinical evaluations from the cardiological e-chart C@RDIONET [17]. In this way, we could identify a cohort of patients without AF history for the prediction of the new onset of AF.

For patients without any AF event in the observation period, we extracted all available ECG exams, while for patients that developed AF, we used all ECGs recorded before the first AF event within a temporal window of 5 years. Note that censored cases, i.e., subjects that did not develop AF, had a minimum follow-up of 5 years required by design. Each ECG was associated with a set of morphological features, automatically extracted by the Mortara devices at the ECG recording. We had access to these features through the cardiological e-chart C@RDIONET.

The AF event was defined linking information from 4 different sources: reports from emergency access or cardiological visits, discharge codes in case of hospitalizations and ECG reports. For each patient, the first AF diagnosis (or atrial flutter) found in one of these data sources was taken as the first AF event. We excluded all patients with an AF event before 2007 or with paced rhythms (i.e., implanted with a pacemaker, PM, with an Implantable Cardiac Defibrillator, ICD, or treated with the Cardiac Resynchronization Therapy, CRT, see the flow chart reported in Fig. S1 of the supplementary materials). Subjects with an AF diagnosis at the first ECG exam or with the AF-event date missing were not included in the analysis.

The unit of observation was the ECG signal. Each ECG was labelled 1 if the corresponding patient will develop AF within 5 years, and 0 otherwise.

### Models' development

The two approaches under study were a deep convolutional neural network (CNN) and an XGBoost model (XGB). XGB is a gradient tree-boosting algorithm that recently has gained great popularity due to its excellent performance in a wide range of problems [18]. A penalized logistic regression model (LR) was used as a benchmark. For all models, the task considered was to predict the probability that a patient will develop AF within five years.

The CNN takes as input the resampled ECG signal, which is a 12 × 5000 matrix (i.e., 12 leads by 10-s duration sampled at 500Hz). The architecture of the CNN is the one used by Scagnetto et al. [19] for AF prediction, which was originally proposed by Goodfellow et al. [20] for a similar purpose, i.e., to classify single lead ECG waveforms as either Normal Sinus Rhythm, AF, or Other Rhythm. The network is composed of 13 blocks, each of which comprises a 1D convolution along the time domain, batch normalization, ReLU activation function and dropout. Notice that, in the computation of convolutions, all channels are used simultaneously, thus cross-lead correlations are automatically leveraged by the model. In blocks 1,6 and 11 there is also a max-pooling layer between ReLU activation and dropout. After the convolutional blocks, there are a global average pooling layer and a soft-max layer, in order to obtain normalized probabilities. All the architecture’s hyperparameters are reported in the appendix (Fig. S2). To train the model we used the cross-entropy loss function and AdamW optimizer [21], with a learning rate of 10^–3^.

The XGB and LR models take as input the wave morphology’s features extracted from the ECG signal by the Mortara devices. These features include the onset and offset of P and T waves and of the QRS complex, the PR and corrected QT intervals, P, T, QRS axis and the cardiac frequency.

To tune the XGB’s parameters, we performed a randomized search over parameters, as described hereafter. For each hyperparameter that we decided to tune, we specified a uniform distribution over the possible parameter values range. Then, we generated a candidate setting of parameters by sampling the pre-specified distributions and we evaluated its performance with a fivefold cross-validation. These steps are repeated 10^5^ times. Finally, the best-performing parameters setting (in terms of AUC) was kept. In this process, we included a set of ECGs (approximately 50.000) solely used for hyper-parameter tuning and not in training/test phases.

In the LR model, we applied an L2 regularization term to reduce overfitting. Therefore, the only parameter of the model is the strength of the regularization term, which we tuned with the same procedure followed for XGB hyperparameters tuning.

The CNN was implemented with PyTorch framework [22] version 1.12.0, while for XGB and LR models we used Scikit-learn 1.0.2 implementations [23]. Python’s version used was 3.10.5. All the code used for this study can be found in the GitHub repository https://github.com/giovabaj/ecg-cnn-xgb-lr.

### Models' evaluation

To assess the ability of the models to discriminate between patients developing/not developing AF, we used the Area Under the Receiver Operating Characteristic Curve (AUC), which is a robust metric of model performance for binary classification, even in the case of imbalanced datasets. Higher AUC values correspond to better performances, with perfect discrimination represented by an AUC value of 1 and an AUC of 0.5 equivalent to a random guess.

To evaluate the models’ calibration, we computed the Integrated Calibration Index (ICI) [24]. Similarly to Cox’s method [25], the ICI is based upon a graphical assessment of calibration, in which the observed binary outcome is regressed on the predicted probability of the outcome, using a locally weighted least squares regression smoother (i.e., the Loess algorithm). Then, a graphical comparison between the smoothed regression line (known as the calibration curve) and the diagonal line with unit slope (that denotes perfect calibration) can be used to assess calibration. However, it is not always easy to interpret graphical calibration curves, mainly because the curve is plotted over the entire range of predicted probabilities and the empirical distribution of these probabilities is frequently not uniform. Thus, a numerical summary of calibration curves is easier to interpret. Specifically, ICI is computed as the weighted average of the absolute difference between the calibration curve and the diagonal line of perfect calibration, where the weights are given by the density function of the predicted probabilities. For a perfectly calibrated model, ICI takes the value of 0, and in general the higher the ICI, the less the model is calibrated. We note that we decided not to use Cox’s intercept and slope because they could be equal to their ideal values of 0 and 1, respectively, while deviations of the calibration curve can still occur around the line of identity [24].

To evaluate the variability of the performance of the trained models, we performed a tenfold cross-validation. In the case of the CNN, 8 sets were used to train the model, 1 to evaluate the model during training and apply early stopping, and the last one to test the model performances on unseen data. Regarding the XGB and LR models, data were split into training and test sets with a 9:1 ratio. In the process of splitting data into folds, in the case of patients with multiple ECGs, we ensured that each patient was present only in one between training, validation and test sets. This is because intra-patient ECGs show a higher degree of correlation with respect to inter-patient ECGs. Thus, without taking into account this detail, the models’ performances would be overestimated. We also made sure that the fraction of positive samples in each fold was as similar as possible to the overall fraction.

### Experimental setting for varying sample sizes/balance

To investigate the dependence of models’ performances on the sample size, we trained the three considered models with increasingly bigger subsets of the dataset. The sizes considered are 1000, 2000, 5000, 10.000, 20.000, 50.000, 100.000 and 150.000 ECGs. The remaining 57.521 ECGs were used to tune hyperparameters for all models.

Another aspect we investigated was imbalance corrections, again for increasingly bigger sample sizes. We repeated the training process described above, but this time balancing the two classes in the training set by random undersampling (RUS), which consists of eliminating a random set of negative ECGs in order to equalize the number of ECGs in each class [26].

To study the effect of class imbalance corrections on models’ performances, we considered a fixed sample size (100.000 ECGs), and we trained the three models with different balancing levels of the training set. The levels considered are 12.5% (corresponding to the original positive fraction), 25%, 37.5% and 50% (perfectly balanced training set). We stress that the test sets used to evaluate the models have always the original positive fraction (12.5%). As before, the method used to balance the training set was RUS, and to estimate the models’ variability we performed a tenfold cross-validation with the same approach described above. Notice that we decided to use a sample size of 100.000 ECGs since we observed that none of the three models showed a substantial improvement with a training size larger than this.

## Results

The final dataset includes 207.521 ECGs, associated to 92.465 subjects. The number of events (i.e. new onset of AF) is 25.857, corresponding to 12.5% of cases. See Table 1 for a descriptive snapshot of the population. Note that the statistical unit of the study cohort is the ECG signal.

**Table 1:** Descriptive features of the dataset. For all the numerical variables median and (1^st^, 3^rd^ quartile) are reported. We compared “Censored” and “Event” populations with Mann–Whitney and Chi-squared tests, respectively for continuous variables and gender. All comparisons were significant (*p*-value < 0.001)

	**Censored**	**Event**	**Overall**
Age (years)	65 (52, 75)	74 (67, 80)	67 (54, 76)
Gender (Male, %)	49	58	50
P axis (degrees)	58 (43, 69)	60 (42, 73)	58 (43, 69)
P onset (msec)	290 (269, 307)	274 (246, 295)	288 (266, 306)
P offset (msec)	407 (388, 422)	391 (361, 413)	406 (385, 422)
PR interval (msec)	163 (148, 182)	176 (157, 199)	164 (149, 184)
QRS axis (degrees)	37 (1, 64)	16 (-22, 53)	35 (-2, 63)
QRS onset (msec)	453 (449, 458)	451 (445, 457)	453 (449, 458)
QRS offset (msec)	550 (543, 558)	551 (545, 563)	550 (543, 558)
QT interval corrected (msec)	408 (395, 424)	420 (404, 439)	409 (396, 426)
T axis (degrees)	54 (36, 68)	59 (34, 78)	55 (36, 69)
T offset (msec)	841 (820, 863)	853 (829, 878)	842 (821, 865)
Heart rate (beats/min)	71 (62, 80)	69 (61, 78)	70 (62, 80)

Compared with censored subjects, patients developing AF were older and more frequently male. These results are not surprising since increasing age is a prominent AF risk factor and the prevalence of AF is lower in women vs. men in most of the real-life study cohorts [27, 28]. Note that we did not include demographic characteristics in the analysis since the objective was to investigate the specific ECG contribution to the prediction. No other remarkable clinical differences are observed in the ECG features.

### Models' evaluation results

In Table 2 we report AUC and ICI values (and corresponding 95% Confidence Intervals, CI) for the three models trained with the biggest sample size considered (150.000 ECGs) and with the original event ratio (no imbalance corrections). We can see that from a discrimination point of view, the CNN model is the best-performing model, with an AUC of 0.799. XGB model is the one with intermediate performance (AUC of 0.74), while LR shows the worst performance (AUC of 0.68). As for the calibration, it can be noticed that there are no substantial differences in the performance of the three models; XGB is the best-performing model with an ICI of 0.008, while the other two models show higher ICI values. In terms of 95% CI, the lower bound of CNN and LR corresponds to the upper bound of XGB.

**Table 2:** Performances in discrimination and calibration of the three models

	**CNN**	**XGB**	**LR**
**AUC**	0.799 (0.794, 0.805)	0.738 (0.732, 0.744)	0.683 (0.678, 0.688)
**ICI**	0.014 (0.01, 0.018)	0.008 (0.006, 0.01)	0.014 (0.013, 0.015)

### Results for varying sample sizes/balance

**Fig. 1:**
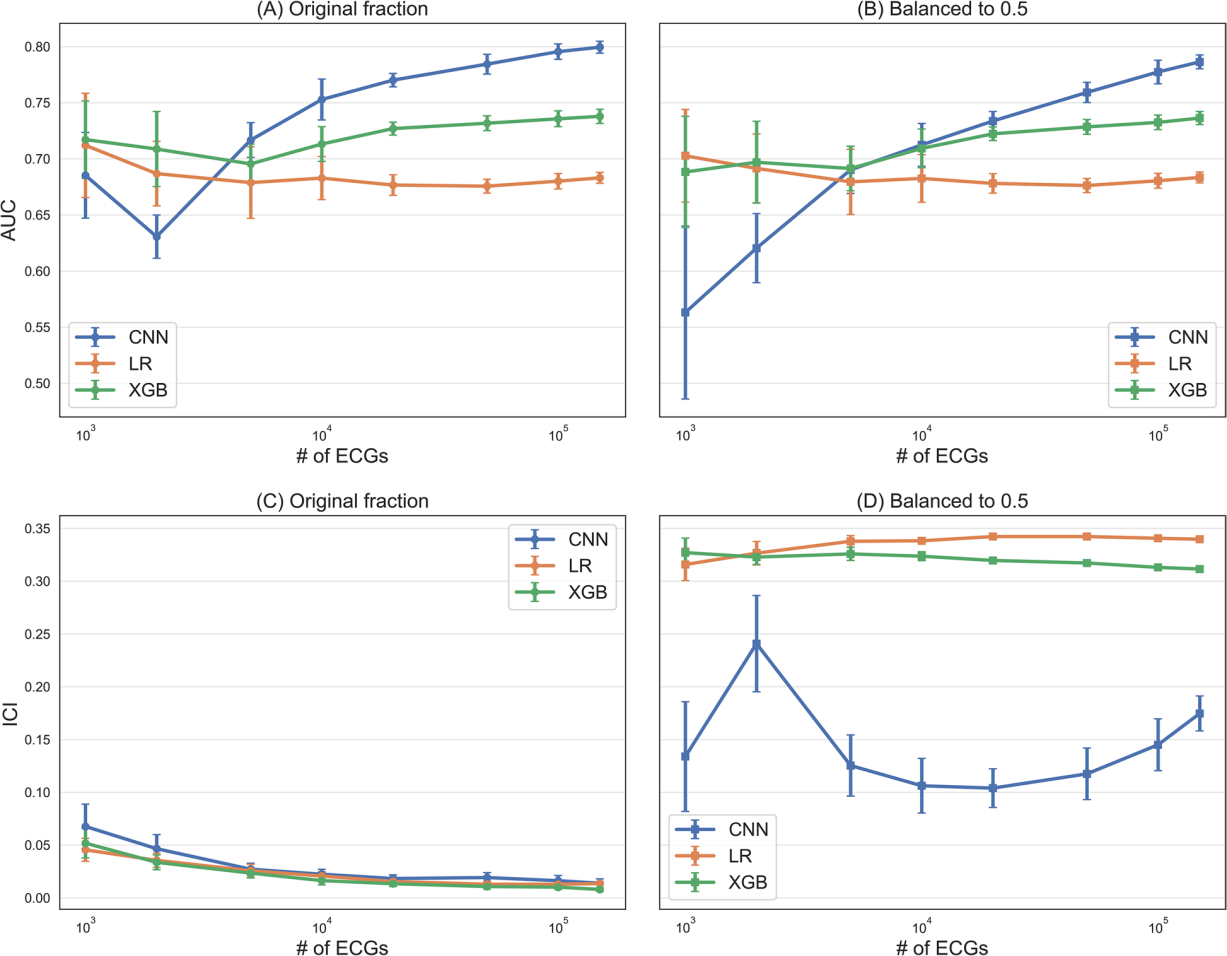
**A** AUC values for the varying sample sizes (original event fraction in the training set). Error bars represent the 95% CI around the mean. **B** AUC values for the varying sample sizes (perfectly balanced training set). **C** ICI values for the varying sample sizes (original event fraction in the training set). **D** ICI values for the varying sample sizes (perfectly balanced training set)

In Fig. 1, we show the dependence of AUC on the sample size for the three proposed models, both in the imbalanced (Fig. 1A) and perfectly balanced (Fig. 1B) cases. We can notice that the model that is most affected by the sample size is the CNN: for small samples the discriminative performances are very low (lower than 0.70), but above 10.000 samples the DL model significantly outperforms XGB and LR, reaching an AUC of 0.80 in the imbalanced case. On the other hand, XGB and LR’s discrimination does not change significantly increasing the sample size, while the most visible effect is the greater variability for small sample sizes, as obviously expected. For these two models the maximum AUC values, obtained with the biggest sample size, are respectively 0.74 and 0.68. Another aspect to note is that balancing the training set with RUS to an event ratio of 0.5 (same number of AF cases and censored samples) does not improve discrimination in any of the models considered, also not for small samples sizes.

In Fig. 1 it is also reported the ICI as a function of the sample size. Figure 1C represents the case where no imbalance corrections were introduced, and we can see that increasing the sample size has the effect of reducing the ICI (i.e., it improves calibration), for all three models under study. In this setting, ICI values range from 0.06 for smaller sample sizes, to approximately 0.01 for the biggest sample size considered. When RUS is applied to balance the training set (Fig. 1D), ICI takes higher values, indicating that models are worse calibrated. The effect is very strong for XGB and LR (ICI values between 0.30 and 0.35 for all the sample sizes considered) and slightly weaker for the CNN model (ICI values between 0.1 and 0.2), but still evident, especially if compared with the imbalanced case.

**Fig. 2:**
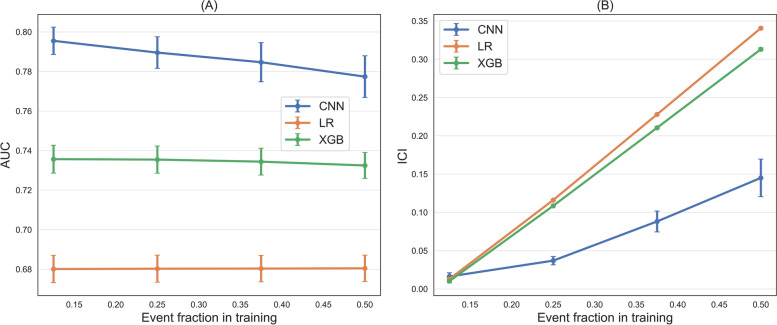
**A** AUC values for the varying event fraction, obtained by balancing the training set with RUS. Error bars represent the 95% CI around the mean. **B** ICI values for the varying event fraction, obtained by balancing the training set with RUS

As regards the effects of imbalance corrections using different event ratios and fixed size of 100.000 ECGs, we found that XGB’s and LR’s discrimination capabilities show very little dependence on the balancing level introduced. This is evident in Fig. 2A, where AUCs are reported for the three models as a function of the event fraction in the training set. Indeed, it can be noticed that XGB and LR models show nearly constant AUC values, respectively of 0.74 and 0.68. As regards the CNN model, also in this case RUS does not allow us to get better discriminative performances, rather AUC slightly decreases as we increase the level of imbalance corrections, approximately from 0.795 to 0.777. Moving to calibration (Fig. 2B), the effect of balancing the training set was very clear: increasing the ratio of positive samples with RUS leads to higher values of the ICI, i.e. to less calibrated models. The effect is very strong for XGB and LR, where ICI values grow linearly from 0.01 to 0.3, and a little weaker for the deep learning model (ICI values from 0.01 to 0.15), but still evident.

## Discussion

In this study, we investigated the use of ECG signals for the development of a predictive model for new-onset AF. This is a critical medical task since the high prevalence of AF particularly in the elderly population and the importance of an early diagnosis of AF for prompt prescription of effective treatments to prevent stroke and systemic thromboembolism.

Two approaches were considered: first, a ML model based on the set of ECG features extracted from the ECG and accessible to clinicians; second, the analysis of the digital ECG traces using deep learning techniques, in a setting of end-to-end analysis. In addition, a logistic regression model based on ECG features was estimated to provide a benchmark for the comparison of results.

As for the analysis of ECG features, for large sample sizes, the XGB algorithm produced a model that outperformed the benchmark in terms of discrimination ability. In particular, the XGB and LR models appeared almost equivalent when the number of observations was lower than 10.000, but for larger sample sizes XGB demonstrated a clear increase in the level of discrimination, resulting however constant in further enlargements of the dataset. In contrast, the CNN model showed a discriminative performance highly dependent on the sample size: to reach a satisfactory result, the DL model required at least 10.000 observations, but for every further increase of the size we observed a correspondent improvement in discrimination. In terms of calibration, no major differences were detected across models when the original fraction of cases was used. In general, we observed better-calibrated predictions for increasing sample size. Our results may suggest that the choice of approach in the analysis of ECG should take into account the amount of data available for the training, preferring more standard models for small datasets, and indicate the well-known ability of DL methods to leverage massive datasets.

The second part of our analysis was focused on the effect of undersampling on models’ calibration. This aspect of the study was stimulated by a recently published work by van den Goorbergh et al. [15] where authors examined the effect of imbalance correction on the performance of standard and penalized (ridge) LR models in terms of discrimination, calibration, and classification. When developing prediction models for a binary outcome with high-class imbalance, undersampling is a standard technique for mitigating the difference in class frequencies in the training phase, with the aim of improving the model’s performance. We analyzed the results of models obtained with different levels of balancing ratios and failed to detect an improvement in discrimination, leading to even worse results in the case of CNN. Besides, imbalance correction caused miscalibrated predictions. Our results are in line with the findings of van den Goorbergh et al. and extend their note of caution in using methods for class imbalance correction in the case for XGB and CNN models. We observe that in our study the CNN resulted more robust compared to XGB and LR to the calibration worsening caused by the imbalance correction, a counter-intuitive finding with respect to what observed by Gou et al. [29].

Concerning the relative performance of our CNN approach with respect to the recent literature that investigated the new onset of AF, Attia et al. considered a set of 649.931 12-lead ECGs of patients ≥ 18 years and applies CNN to identify the electrocardiographic signature of future AF developed within one month from ECG examination (8.4% of the cohort). They obtained a very accurate model (AUC 0.90 [0.90–0.91]), but the sample size and the time-frame prediction period are clearly very different from ours. Another relevant study was carried out by Ragunath et al. [12], in which authors analyzed 1.6 M 12-lead ECGs from patients aged 18 years or older in order to identify individuals at risk of developing AF within 1 year. Training a CNN using only ECG traces as input, they were able to predict the new onset of AF with an AUC of 0.83 (95% CI, 0.83—0.84). Although the sample size and observational period are different from ours also in this case, the performance is comparable with our findings (Table 2). No measures of calibration were reported in those works.

Our study has some limitations. First, we could not validate our findings in an external validation cohort that represents one of the most critical steps in the development of machine learning models in medicine, a context where internal validation is not considered sufficiently conservative [30]. Second, for AF subjects we only considered ECG exams no further than 5 years before the date of AF diagnosis. We set such constraints because based on clinical knowledge, AF individuals are unlikely to show predictive signs of the condition earlier than 5 years. The methodological choice is also in line with previous clinical scores and predictive models that are usually evaluated at a time horizon of 5 years of follow-up [3]. Third, in order to simplify the prediction task, we did not take into account the time-to-event in disease onset. A very recent research carried out by Khurshid et al. [31] has highlighted the potential of CNN for the prediction of the time-to-incident AF and obtained very accurate predictions (5-years AUC 0.823 [95% CI, (0.790—0.856]). One of the advantages of the time-to-event data is the possibility to evaluate the accuracy of the model for any time frame from the baseline.

Another possible limitation was the choice of the method to correct the class imbalance, as RUS is a very *naïve* approach. The main obstacle here was to deal with entire signals. For example, a commonly used method that has shown good results in various applications is the synthetic minority oversampling technique (SMOTE) [32]. SMOTE is an oversampling approach that creates new, synthetic samples interpolating the original minority class samples. This method and its variations were developed for tabular data, but an extension in the case of signals is not straightforward. Some methods to generate synthetic ECG signals were recently proposed [33–36], but it was out of the scope of this work. Finally, the fact that only standard ECG features were used for the XGB approach is a clear limitation, considering that several ECG-engineered features were shown to be highly predictive for AF detection [4] and AF risk prediction [37, 38]. We expect that including this kind of feature engineered from the ECG signal could improve XBG performances. However, we want to highlight that we limited on purpose to the features automatically extracted by electrocardiographs since we wanted to consider a setting as simple as possible, where only the ECG exam is required so that the prediction process can be easily automated without the need for feature engineering by experts.

Future developments of the present study will include the integration of standard tabular information (sex, age, clinical information) as predictors in addition to ECG traces. According to the findings of recent studies [37, 39], new tools are emerging to combine deep representations of data obtained from convolutional neural networks (in substitution to human feature engineering) with electronic health records tabular information. In our opinion, such methodologies intended to integrate heterogeneous data sources could have great potential, in particular if extended to time-to-event data analysis, since employing deep learning models represents the most promising and feasible approach to operate in ultrahigh dimensional settings, as the case of ECG waveforms. Another future development of this work is the application of explainability techniques to investigate the prediction mechanism of our models. Indeed, clinical interpretability is a fundamental step in order to build predictive tools for clinical usage, which is one of our main goals for the future.

## Conclusions

The deep learning model under study showed a discriminative performance highly dependent on the sample size, outperforming the two approaches considered based on the signal’s extracted features only above a certain sample size threshold. This result suggests that the choice of approach in the analysis of ECG should be based on the amount of data available, preferring more standard models for small datasets.

Imbalance corrections with a random undersampling approach did not lead to better discrimination performance, rather to an evident drop in models’ calibration. This finding indicates that imbalance correction methods should be avoided when developing clinical prediction models.

## Supplementary Information

**Additional file 1.**

## Data Availability

Data are from administrative databases of the Cardiovascular Centre of Trieste. The owner of the data is Azienda Sanitaria Universitaria Giuliano Isontina (ASUGI). We are not allowed to share data publicly, since sensitive information about patients is contained. Analyzed data are linked and anonymized before being passed to the analysts. The person in charge of data control for the government is: Dr. Andrea Di Lenarda, Director of Cardiovascular Center, University Hospital and Health Services of Trieste, Trieste, Italy ccv@asugi.sanita.fvg.it. Data can be requested for researchers who meet the criteria at sri@asugi.sanita.fvg.it, SC Ricerca e Innovazione Clinico Assistenziale (ASUGI), Via Giovanni Sai 1—3, 34128 Trieste, Italy.

## Abbreviations

AF: Atrial fibrillation
AI: Artificial intelligence
AUC: Area under the receiver operating characteristic curve
CNN: Convolutional neural network
DL: Deep learning
ECG: Electrocardiogram
ICI: Integrated calibration index
LR: Logistic regression
ML: Machine learning
RUS: Random undersampling
SMOTE: Synthetic minority oversampling technique
XGB: Extreme gradient boosting
