# Supplementary material for: Comparison of discrimination and calibration performance of ECG-based machine learning models for prediction of new-onset atrial fibrillation
Source: BMC Med Res Methodol. 2023 Jul 22;23:169. doi: 10.1186/s12874-023-01989-3 (PMC10363301; doi:10.1186/s12874-023-01989-3)
Supplement: Supplementary file 1 — Additional file 1. [file 12874_2023_1989_MOESM1_ESM.zip › Supplementary_materials.docx]

**SUPPLEMENTARY MATERIAL**

**Hyperparameters tuning**

Hyperparameters are parameters that are not directly learnt within estimators. We optimized hyperparameteres with RandomizedSearchCV class of scikit-learn package. The idea is to sample a given number of candidates from a parameter space with a specified distribution. The hyperparameters with the best cross-validation AUC are then chosen.

Hereafter we report the best performing hyperparameters for XGB and LR models. Hyperparameters not reported were not tuned and used with their default value. The distributions from which we sampled candidate settings are given in the script “hyperparameters_search.py”, included in the Github repository of the paper.

XGB:

- colsample_bytree: 0.9994406226497948
- gamma: 4.575305537258395
- learning_rate: 0.02631221490906356
- max_delta_step: 0
- max_depth: 9
- min_child_weight: 7
- n_estimators: 275
- subsample: 0.6089510933763641

LR:

- C: 30.73202411565708
- penalty: “l2”

**Feature importance**

We report the regression coefficient of the logistic regression model trained with the biggest training size (150.000 ECGs) and without random undersampling (RUS). Notice that 10 different models were trained with this setting, since we applied 10-fold cross-validation. In Table S1 we report the coefficients of just one of the trained models. We verified that the regression coefficients were very similar for all the other cross-validation models.

Table S1. Standardized regression coefficients of the logistic regression model.

| **Features** | **Betas** |
| --- | --- |
| T offset | 0.349627 |
| P offset | -0.317819 |
| Heart rate | 0.197899 |
| QRS axis | -0.171053 |
| T axis | 0.084284 |
| QRS onset | -0.062184 |
| P axis | 0.049434 |
| QRS offset | 0.048984 |
| P onset | -0.035136 |
| PR interval | 0.015128 |
| QTC interval | 0.006014 |

As regards XGBoost, we report the feature importance values directly estimated by the package (Table S2). As before, we consider only one of the models trained with the maximum training size and without RUS. We verified that all the cross-validation models trained with the same setting shared very similar feature importance.

Table S2. Feature importance for XGBoost model.

| **Features** | **Importance** |
| --- | --- |
| P offset | 0.1889 |
| QTC interval | 0.1132 |
| T axis | 0.1022 |
| P onset | 0.0999 |
| P axis | 0.08982 |
| QRS axis | 0.07911 |
| T offset | 0.07035 |
| QRS onset | 0.06750 |
| PR interval | 0.06597 |
| QRS offset | 0.06307 |
| Heart rate | 0.06015 |

**Legends**

Figure S1. Flow chart of the study cohort

Figure S2. CNN architecture
