# Supplementary figures and images for: Comparison of discrimination and calibration performance of ECG-based machine learning models for prediction of new-onset atrial fibrillation
Source: BMC Med Res Methodol. 2023 Jul 22;23:169. doi: 10.1186/s12874-023-01989-3 (PMC10363301; doi:10.1186/s12874-023-01989-3)

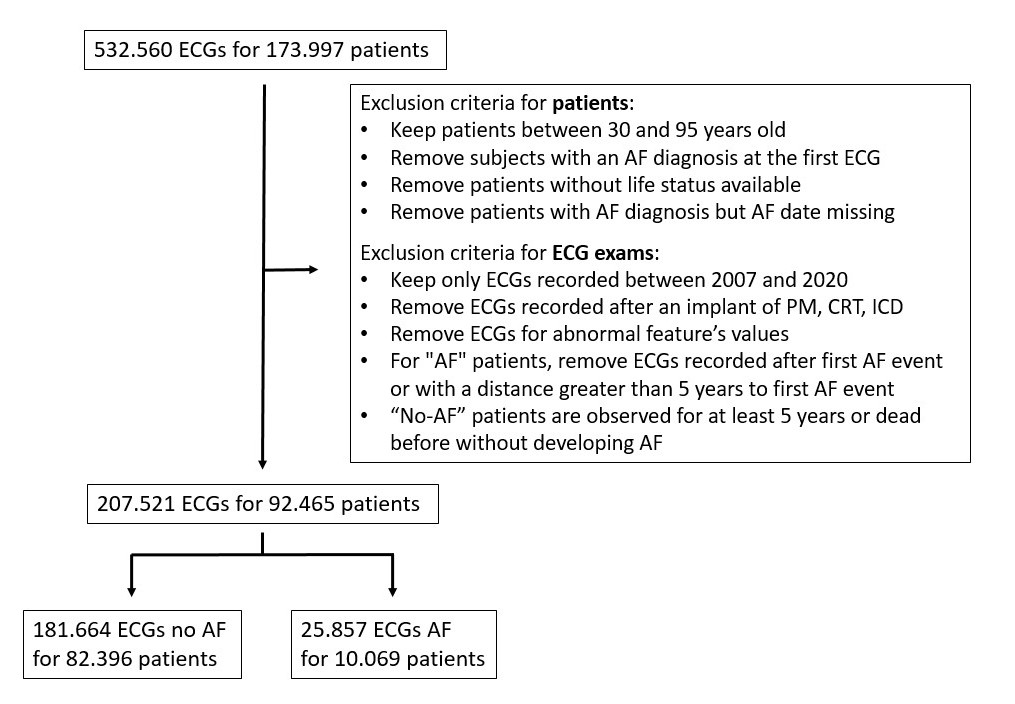

Supplement: Supplementary file 1 — Additional file 1. [file 12874_2023_1989_MOESM1_ESM.zip › FigureS1.jpg]

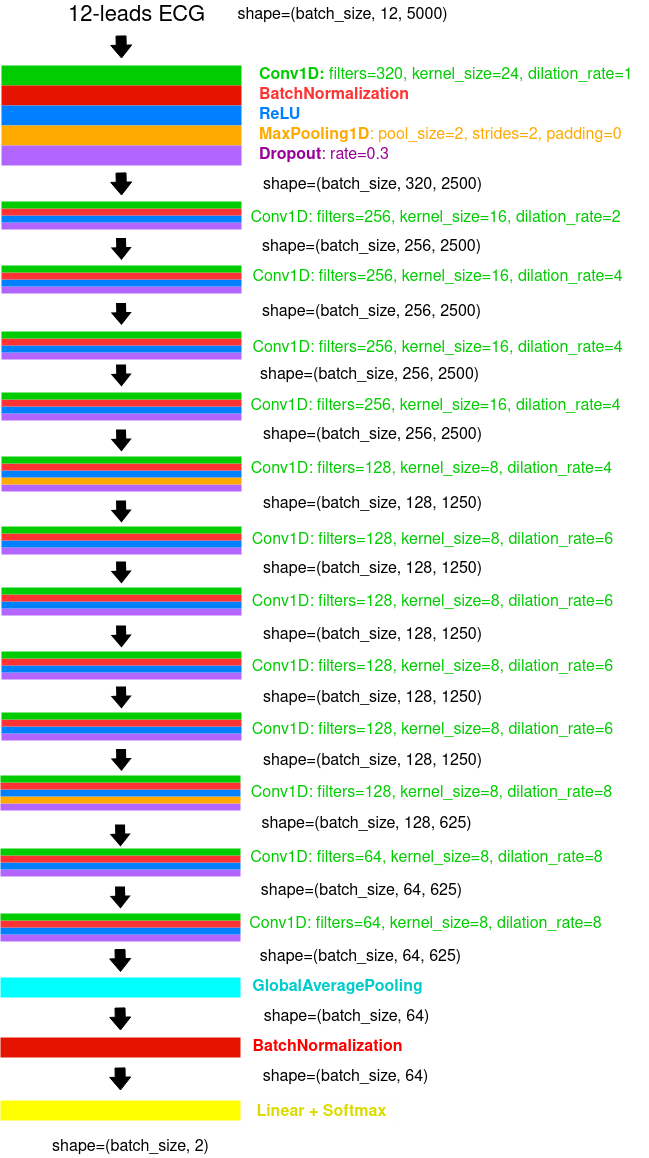

Supplement: Supplementary file 1 — Additional file 1. [file 12874_2023_1989_MOESM1_ESM.zip › FigureS2.png]
